# Supplementary material for: Disorder engineering and conductivity dome in ReS2 with electrolyte gating
Source: Nat Commun. 2016 Aug 8;7:12391. doi: 10.1038/ncomms12391 (PMC4979068; doi:10.1038/ncomms12391)
Supplement: Supplementary Information — Supplementary Figures 1-11, Supplementary Notes 1-12, Supplementary Methods and Supplementary References [file ncomms12391-s1.pdf]

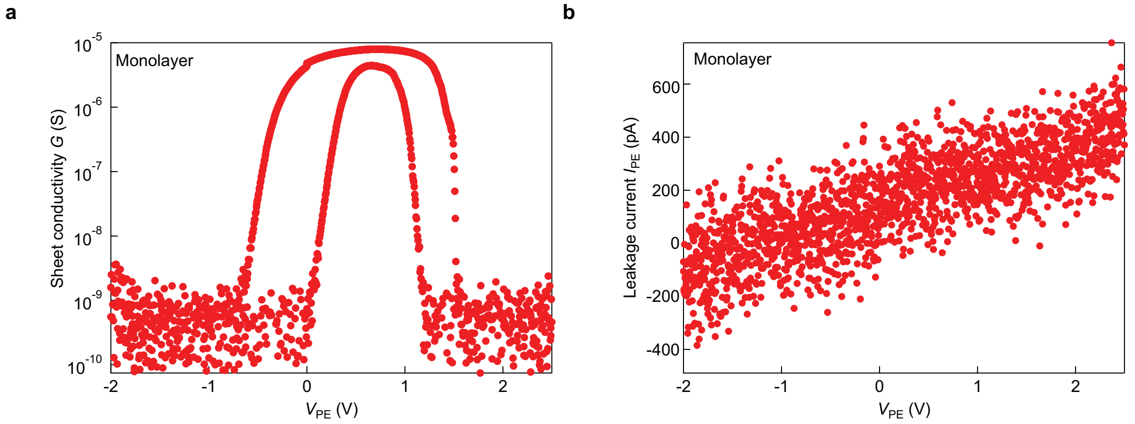

Supplementary Figure 1. Comparison of channel and leakage currents in the monolayer ReS_2 device. a, Sheet conductivity G as function of V_{PE} in monolayer ReS_2. **b,** Leakage current I_{PE}, recorded in the same time.

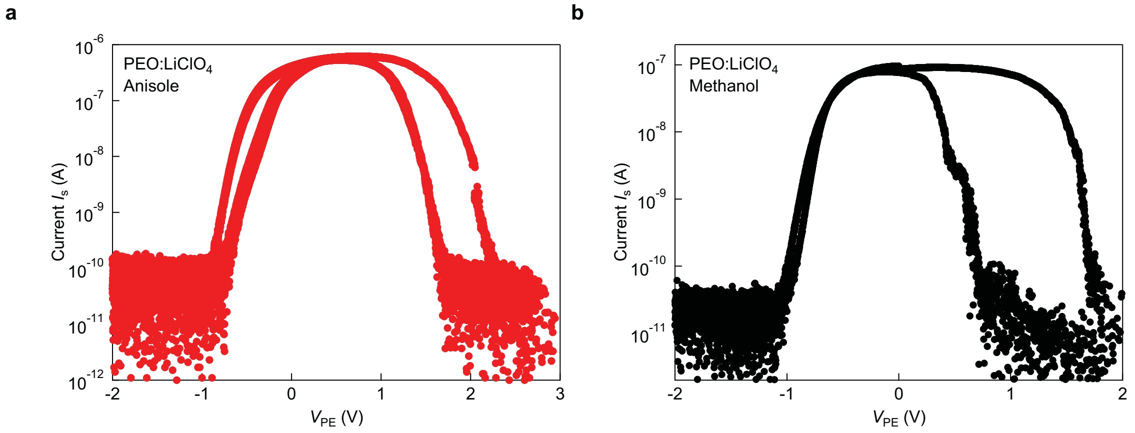

Supplementary Figure 2. Transport in monolayer ReS_2 EDLT with electrolytes based on $LiClO_4$. a, b I_s-V_{PE} sweeps of two representative devices with a polymer electrolyte based on $LiClO_4$.

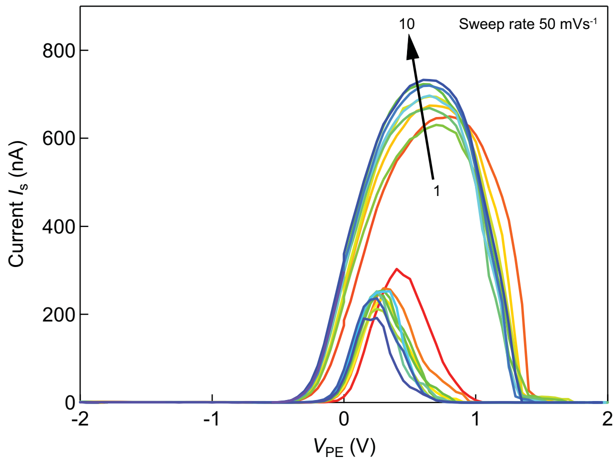

Supplementary Figure 3. Cycling of V_{PE} in monolayer ReS_2. Color code from red to blue corresponds to sweep number from 1 to 10.

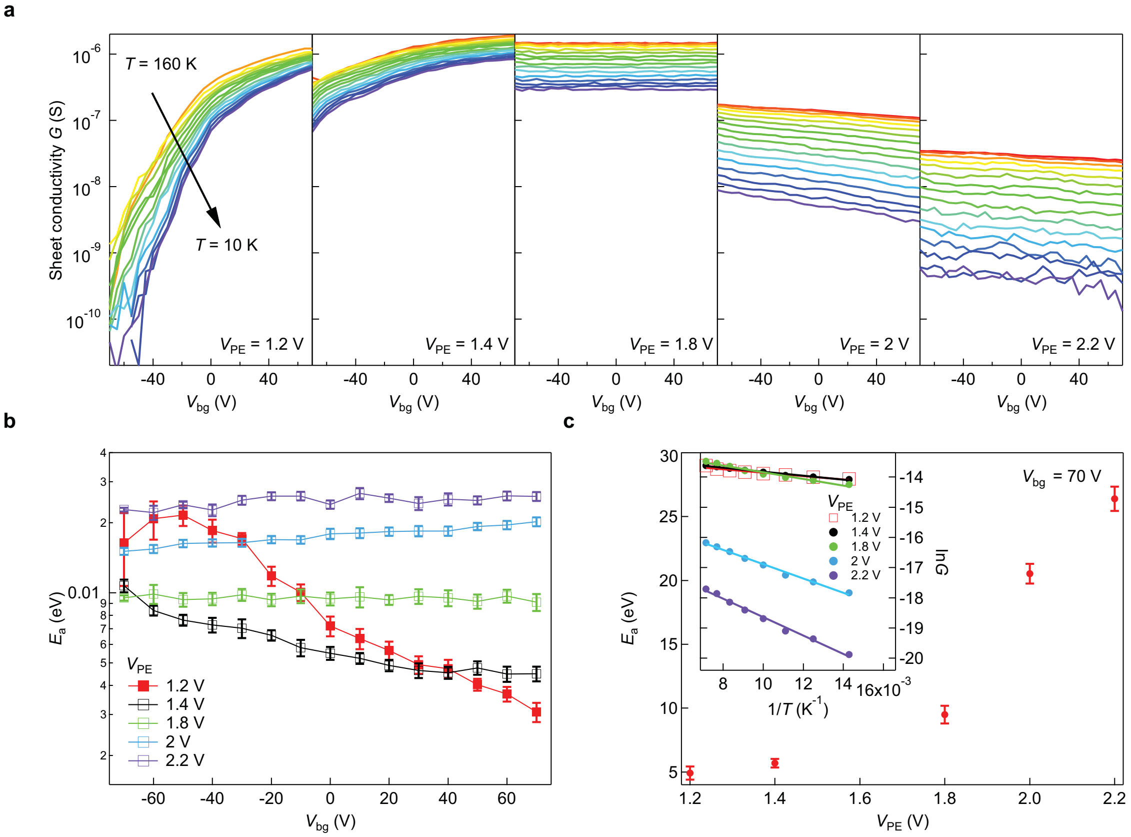

Supplementary Figure 4. Monolayer ReS_2 with polymer electrolyte on SiO_2 substrate. a, Series of 5 consecutive cooldowns for different values of applied V_{PE}. Color code from red to blue corresponds to temperatures from 160K to 10K. **b**, Activation energy E_a in the logarithmic scale as a function of V_{bg} and V_{PE}. **c**, Activation energy E_a at V_{bg} = 70V as a function of V_{PE}. Inset - corresponding Arrhenius plots from which E_a was extracted. Solid lines correspond to the linear fits to the model $G(T) = G_0 e^{-E_a/k_B T}$. Error bars in **b** and **c** originate from uncertainties in linear fits of ln$G$ as a function of 1/T.

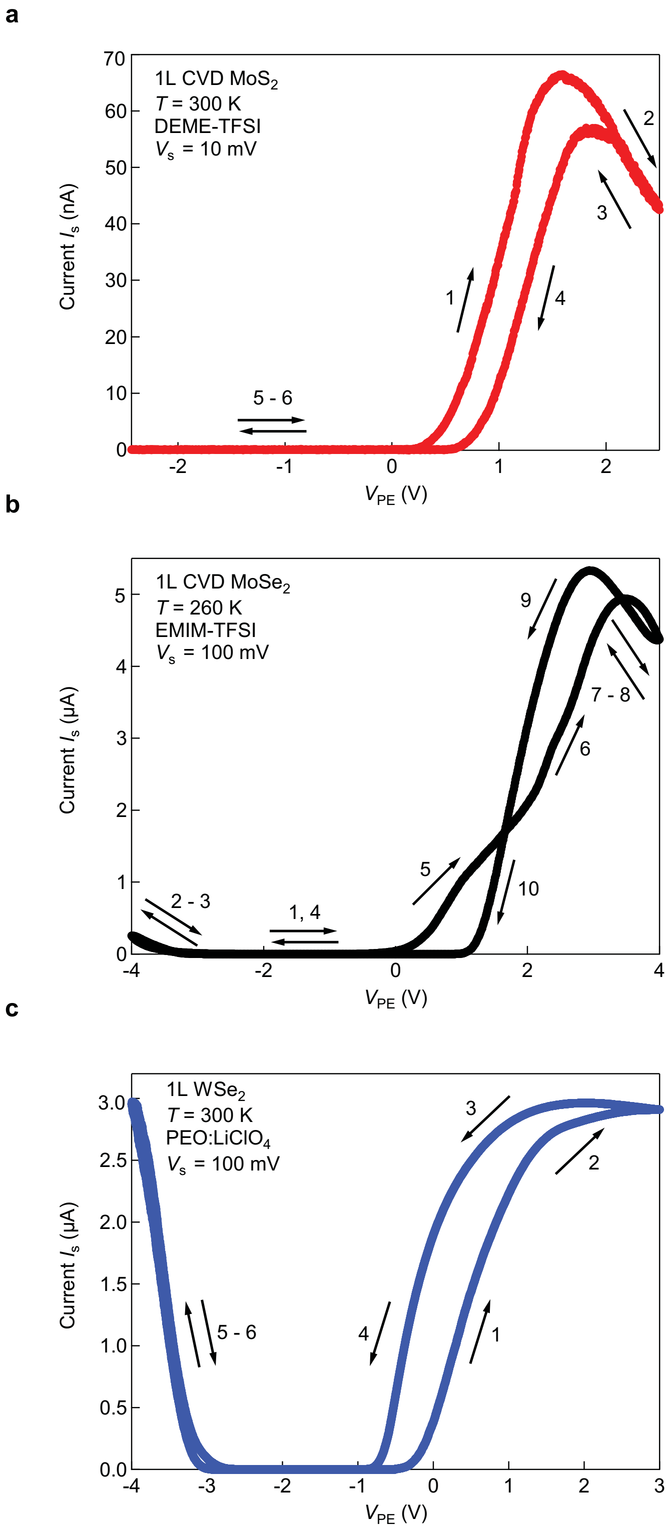

Supplementary Figure 5. EDLT based on monolayer TMDCs. a, Current I_s as a function of ionic liquid DEME-TFSI potential V_{PE} for monolayer CVD MoS_2. **b,** The same for monolayer CVD $MoSe_2$, here [EMIM]-[TFSI] was used as PE. **c,** The same for monolayer WSe_2. PEO:$LiClO_4$ was used as PE. Arrows are showing the sweep direction.

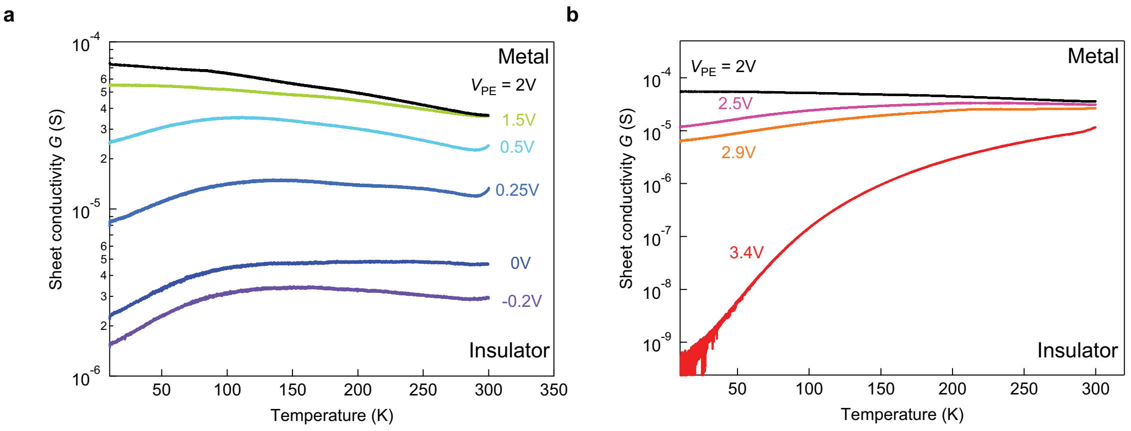

Supplementary Figure 6. Insulator-metal-insulator sequence in 10 nm thick ReS_2. a,b Sheet conductivity *G* as a function of *T* for different values of V_{PE} in 10 nm thick flake, discussed in the main text, Figure 3(b) and Figure 4.

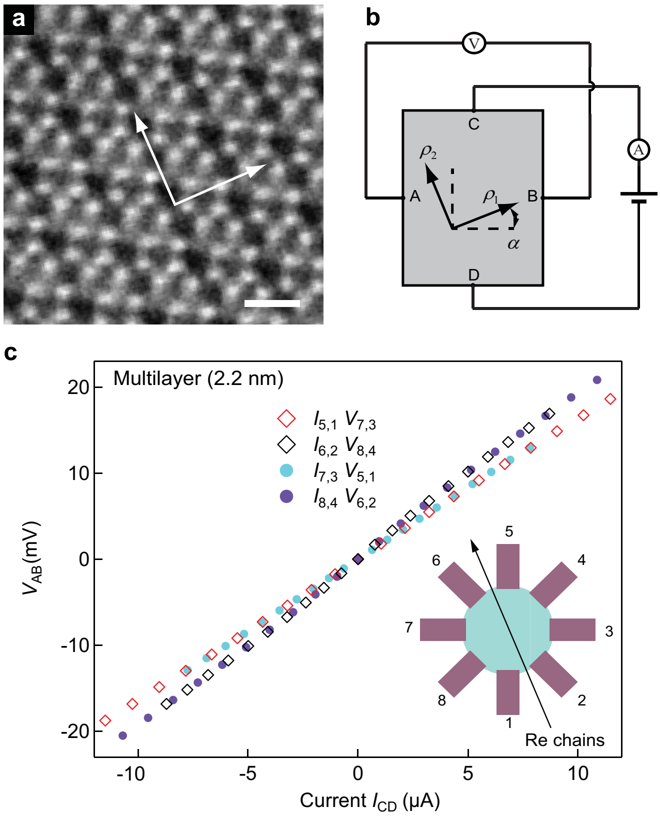

Supplementary Figure 7. Anisotropy in trilayer ReS_2. a, Scanning Transmission Electron Microscopy (STEM) micrograph of multilayer ReS_2, arrows are showing orientation of easy and hard axis. Scale bar - 500 pm. **b**, Schematic of reciprocal measurements, where the lattice is arbitrarily oriented with respect to current flow. **c**, Reciprocal measurements at zero magnetic field. Inset: schematic of the device with the contact pairs labeled. The arrow shows the easy axis direction, corresponding to high conductivity direction.

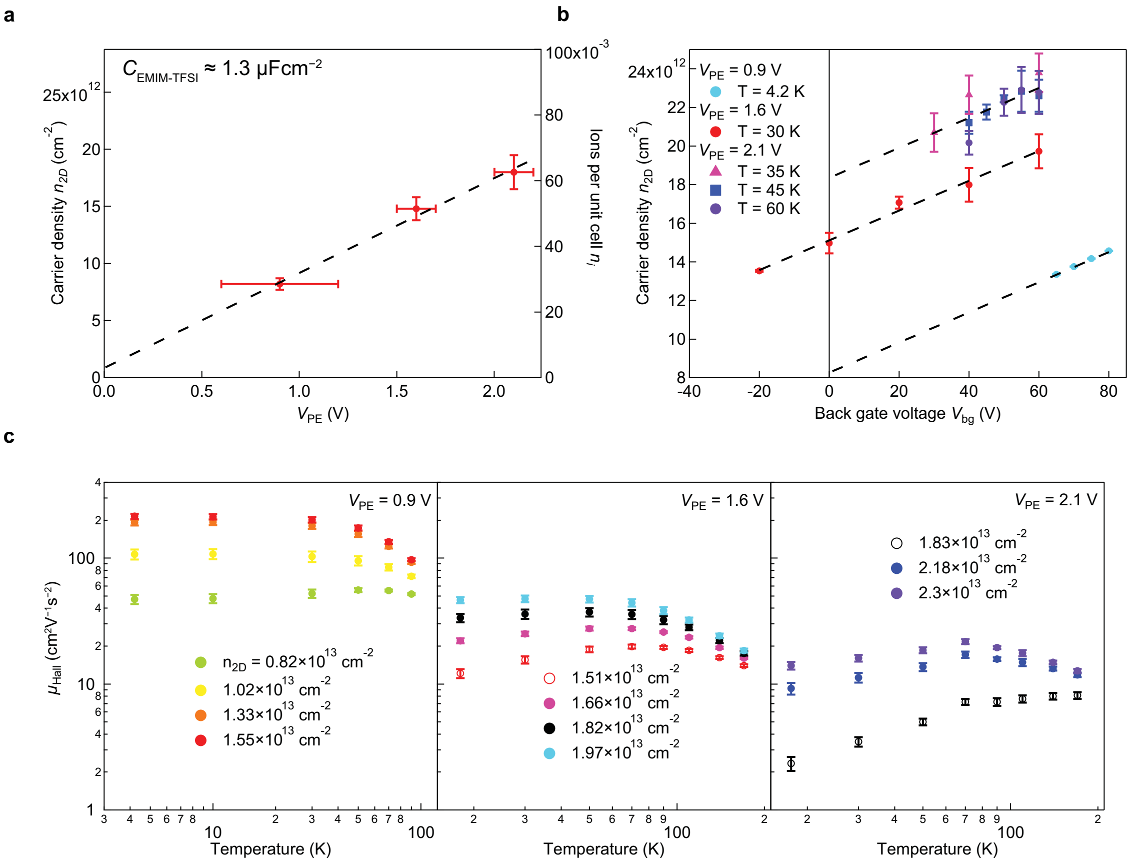

Supplementary Figure 8. Determination of the polymer electrolyte gate capacitance and mobility measurements. a, Carrier density n_{2D} as a function of V_{PE}. Error bars in carrier density determination originate from uncertainties in Hall effect measurements. Error bars in V_{PE} determination take into account hysteresis. **b,** Carrier density as a function of V_{bg} for 3 cooldowns. Error bars originate from uncertainties in Hall effect measurements. Dashed black lines are linear fits for n_{2D} as a function of V_{PE}. **c,** Hall effect mobilities at different n_{2D} and V_{PE} as a function of temperature. Error bars originate from uncertainties in Hall effect carrier density measurements and conductivity extraction.

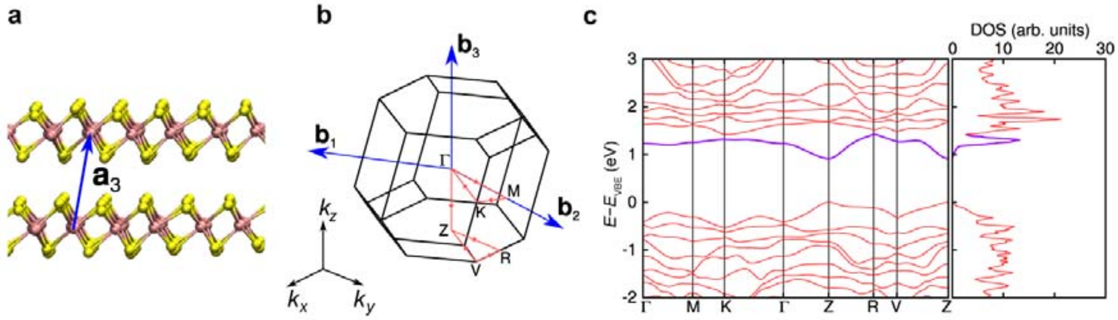

Supplementary Figure 9. Electronic structure of bulk ReS_2 from ab initio calculations. a, Ball-and-stick representation of the atomic layer stacking of bulk ReS_2. Primitive vector $\mathbf{a}_3$ is illustrated. **b**, Brillouin zone, primitive vectors $\mathbf{b}_1$, $\mathbf{b}_2$, and $\mathbf{b}_3$ of reciprocal lattice, and k-point path for band structure calculation (red lines). **c**, Energy bands calculated along high-symmetry directions connecting the vertices defined in (b) and k-integrated density of states (right panel). The lowest energy conductance band as well as its contribution in the DOS are highlighted in purple.

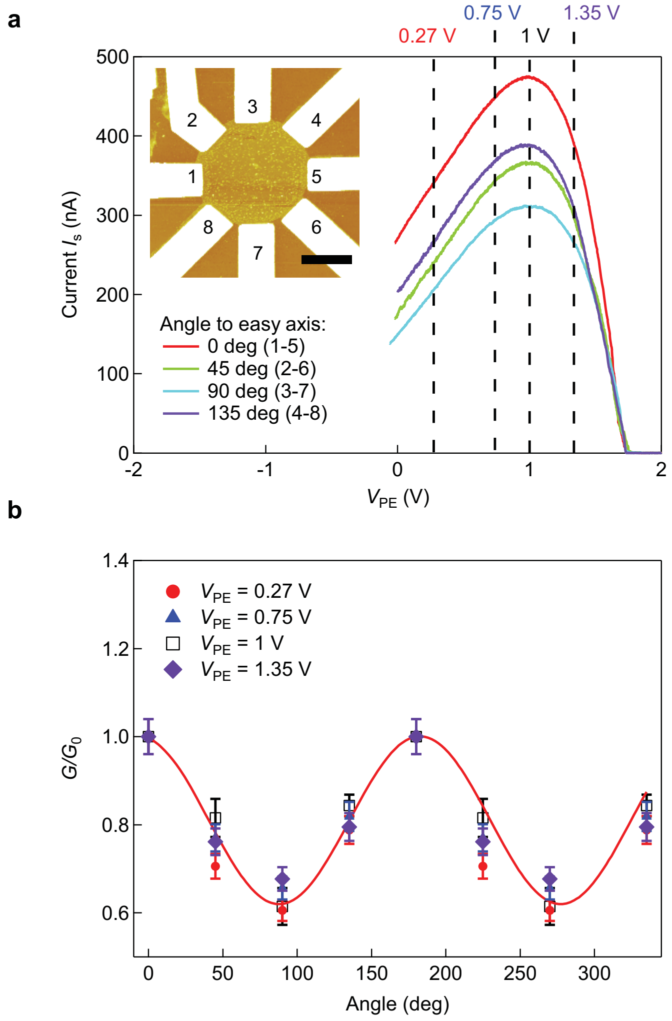

Supplementary Figure 10. Anisotropy in monolayer ReS_2. a, Lattice orientation-dependent measurements of monolayer ReS_2. Inset - AFM micrograph of the device, contacts are marked with numbers. **b,** Reduced conductivity G/G_0 at different doping levels, red line: sinusoidal fit. Error bars originate from the ratio, calculated for different sweep directions.

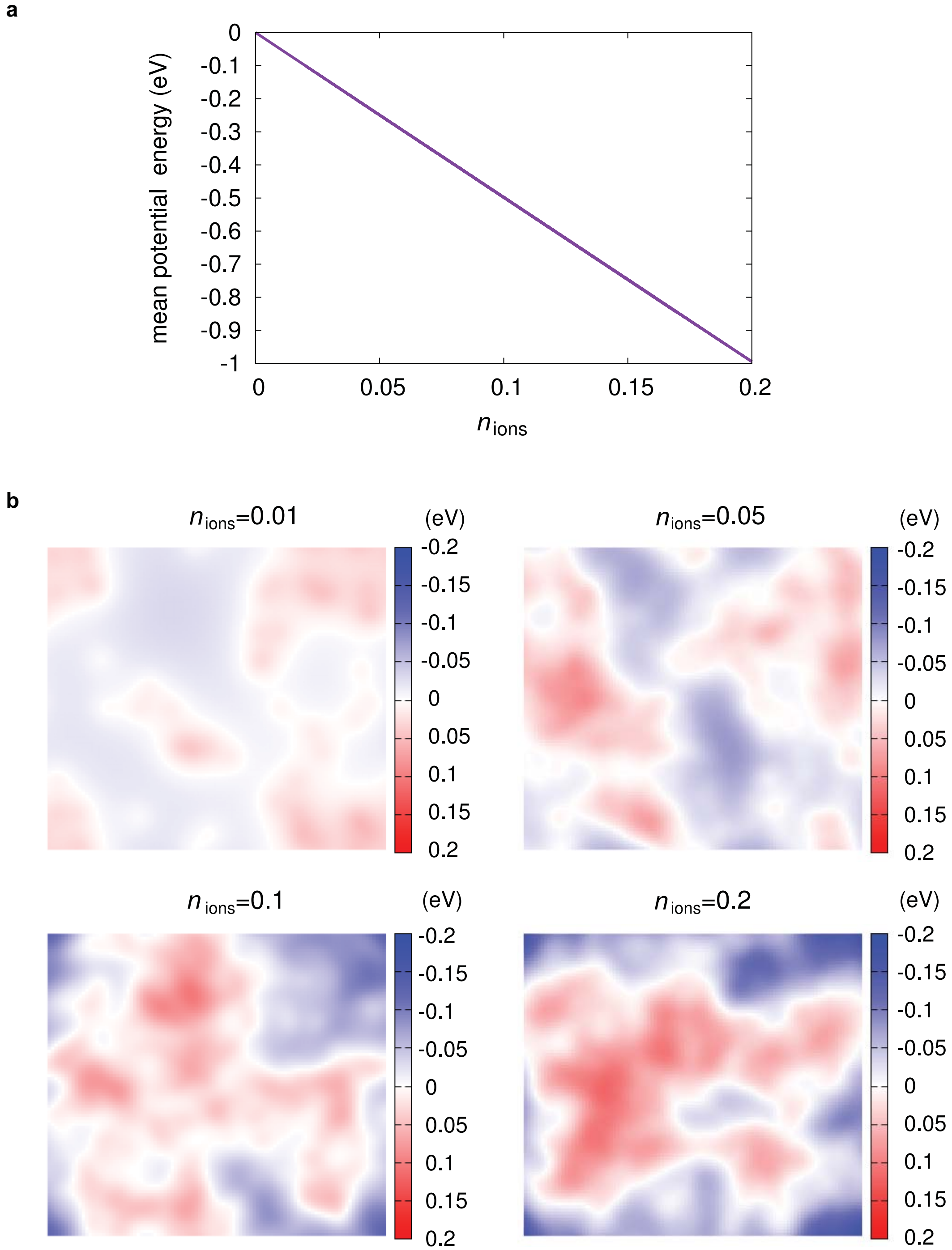

Supplementary Figure 11. Distribution of the electrostatic potential energy calculated for the effective rectangular lattice. a, Spatially averaged values of the electrostatic potential energy as a function of ionic concentration n_{ions}. **b,** Maps representing the fluctuation of the potential energy with respect to the mean spatial value. The four panels refer to four different concentrations n_{ions}.

Supplementary Note 1: Monolayer ReS_2 EDLT - four probe measurements and leakage current

We have measured the sheet conductivity G as a function of V_{PE} for the monolayer device from the main text (discussed in Figure 1), and present it here on Supplementary Figure 1a. This curve reproduces the conductivity dome, visible in current field-effect curves and demonstrates that the observed behavior is not a contact effect. On Supplementary Figure 1b we present the leakage current, recorded simultaneously with the drain current. The values, not exceeding 700 pA, indicate the absence of electrochemical reactions and flake degradation.

Supplementary Note 2: Devices with different electrolytes

The devices from the main text were measured using [EMIM]-[TFSI]. To make sure that the observed behavior is not an artifact related to the use of a specific polymer electrolyte, we employed a different polymer electrolyte based on $LiClO_4$. The same behavior and dome-like shape was observed in I_s-V_{PE} sweeps, as shown on Supplementary Figure 2. The sweep rate was 1 mVs^{-1} in both cases.

We notice that the difference between size of ions used in EMIM-TFSI and $LiClO_4$ is significant. Under the application of positive voltages, $[EMIM]^+$ and Li^+ ions are accumulated on the surface of ReS_2 flake, while the ions with negative charge are also present, but in a smaller amount. Thus the observed suppression of conductivity at high carrier densities could be explained by the presence of ions of varying size, which creates electrostatic disorder in the vicinity of the conductive channel.

Supplementary Note 3: Cycling of V_{PE} in monolayer ReS_2 ELDTs

To rule out degradation during cycling or detrimental influence of ions on our device performance we have performed cycling of V_{PE} 10 times at elevated sweep speed (50 mVs^{-1}) in the monolayer ReS_2 EDLT. The measurements were performed at 300K. These measurements revealed that current stays at the same level, as shown on Supplementary Figure 3. Color code from red to blue corresponds to cycles from 1 to 10.

Supplementary Note 4: Temperature-dependent measurement of monolayer ReS_2 on SiO_2 substrates with PE

Similarly to the case of monolayer ReS_2 on HfO_2 substrate, discussed in main text, we have performed temperature-dependent studies of devices on SiO_2 substrates. In general, the substrate should not play a major role here. However, to rule out the possibility of material damage or other artifacts introduced during transfer, we have performed temperature measurements on flakes exfoliated onto 270 nm SiO_2 substrates. On Supplementary Figure 4a, a series of cooldowns at different V_{PE} are presented. Below 160K we use the back gate to modulate the carrier density. The scale is kept the same to track the evolution of conductivity with V_{PE}. The same analysis as for the case of device from the main text (Figure 2) of Arrhenius curves was employed in the range of $80 - 140$ K to extract the activation energy, which is plotted as a function of back gate voltage V_{bg} in the logarithmic scale on Supplementary Figure 4b. The first cooldown at $V_{PE} = 1.2$ V around the band edge illustrates the standard behavior of activation energy, where decrease of E_a with increasing V_{bg} is observed. The next cooldown already shows more flat curve with increase of E_a. Furthermore, a gradual increase of E_a with increasing V_{PE} is observed.

On Supplementary Figure 4c we plot the representative activation energy at $V_{bg} = 70$ V as a function of V_{PE}. In the inset of Supplementary Figure 4c, we present ln G as a function of $1/T$. Curves from red to green, having almost the same level of conductivity, show a gradual increase of slope (and consecutively activation energy), while the increase continues on the right-hand side of the conductivity dome with the drop of conductivity. This is in agreement with the monolayer device on an HfO_2 substrate presented in the main text. For this device, temperature measurements were performed up to lower values of conductivity, where E_a reaches the value ~27 meV.

Supplementary Note 5: Other monolayer TMDCs at high carrier densities

In this section we discuss conductivity domes in conduction band of EDLTs based on monolayer MoS_2, $MoSe_2$ and WSe_2. MoS_2[1] and $MoSe_2$ were grown using the chemical vapor deposition (CVD) method while WSe_2 was exfoliated from bulk CVT crystals. On Supplementary Figure 5 we show sweeps of ionic liquid (IL) or PE voltages for these materials. Hysteresis is present in each curve and depends on the IL or PE used as well as the speed, temperature and direction of sweep. The conductivity dome is apparent in the conduction band. After the dome at high electron doping in case of WSe_2 we could observe insulating behavior in temperature, as reported before.[2]

Although this resembles the situation with monolayer ReS_2, complete conductivity suppression could not be achieved in MoS_2, $MoSe_2$ or WSe_2. Other important differences include the metallic state, which could be observed in monolayer WSe_2[2] before the conductivity dome. The same behavior was observed in monolayer $MoSe_2$, detailed study will be published elsewhere. We compare our results with literature for bulk material and find that thicker flakes of MoS_2,[3,4] $MoSe_2$[4] and WSe_2[5] at high electron doping exhibit band-like transport (and eventually superconductivity in mono- and multilayer MoS_2[3,4,6] and multilayer $MoSe_2$[4]). Thus we conclude that monolayer ReS_2 behaves uniquely in the family of monolayer TMDCs under ionic gating, while multilayer ReS_2 stands apart from other semiconducting multilayer TMDCs due to the emerging insulating state at high carrier densities, discussed on Figure 3-4 of the main text.

Supplementary Note 6: Cooling curves for multilayer (10 nm thick) ReS_2

The summary of several cooldowns of 10 nm thick ReS_2 flake is presented in Figure 3(b) in the main text. The details of these measurements are presented on Supplementary Figure 6a, b. The V_{PE} is applied at room temperature, the current is stabilized and the cooldown is performed with constant speed (1K/min).

Supplementary Note 7: Anisotropy and Hall effect in three-layer ReS_2

Prior to Hall effect (HE) measurements, we discuss an important feature, which could influence the HE interpretation: the anisotropy of ReS_2. Supplementary Figure 7a shows a scanning transmission electron microscopy (STEM) micrograph of multilayer ReS_2. The lattice is distorted, which manifests itself in chains of Re atoms, which correspond to the easy axis[7] - the axis of low resistance. We have performed measurements in two-terminal directional monolayer structures (see also Supplementary Note 11). However, there is another method to determine the conductivities along the easy and hard axis, without knowing their orientations *a priori*. It is of interest in samples where the crystal orientation is unknown and sheds light on the interpretation of HE measurement in ReS_2. Supplementary Figure 7 shows such an analysis on a "directional" three-layer (3L) ReS_2 device (Supplementary Figure 7c, inset).

In anisotropic materials, Hall voltages have a magnetic field-independent component. If we consider the crystal orientation schematically shown on Supplementary Figure 7b, the transverse voltage $V_{A,B}$ in response to an applied current $I_{C,D}$ is given by:[8]

$$V_{A,B} = \left(-\frac{1}{2}(\rho_1 - \rho_2)\sin 2\alpha + C_H B \right) I_{C,D} \tag{1}$$

where α is the angle between the current flow and one of the main crystalline directions, ρ_1 and ρ_2 are the resistivities along each axis, C_H is the Hall coefficient and B is the magnetic field magnitude. When measuring the carrier density, the anisotropic contribution manifests itself as an offset voltage at zero magnetic field. At zero magnetic field, equation (1) is π-periodic and reciprocal measurements (swapping the current-carrying and voltage-reading pairs) are equivalent, as can be seen in Supplementary Figure 7c. But having eight electrodes, we have two sets of four electrodes rotated by 45°, which allow us to obtain a system of two independent equations that can be solved to determine $(\rho_1 - \rho_2)$ and 2α:

$$V_{3,7} = \left(-\frac{1}{2}(\rho_1 - \rho_2)\sin 2\alpha \right) I_{5,1} \tag{2}$$

$$V_{4,8} = \left(-\frac{1}{2}(\rho_1 - \rho_2)\cos 2\alpha \right) I_{6,2} \tag{3}$$

Having solved for 2α we can now assign the direction of the easy axis (here $\alpha \approx 20$ °, Supplementary Figure 7c). Finally, we perform van der Pauw (vdP) measurements. It is known[9] that for anisotropic material the solution of the vdP equation gives $R_s = \sqrt{\rho_1 \rho_2}$. With this and $(\rho_1 - \rho_2)$ we can calculate the absolute resistivity along each of the two axis. Moreover, the resistivity ratio that we can now calculate contains no errors due to contact resistances. We find a value of $\rho_1/\rho_2 \approx 0.57$.

Furthermore, we have performed HE measurements. We extracted the capacitance of [EMIM]-[TFSI], the carrier density as a function of V_{PE} is shown on Supplementary Figure 8a. The value of $C_{\text{EMIM-TFSI}}^{\text{Hall}} \approx 1.3 \mu\text{F}\cdot\text{cm}^{-2}$ is slightly lower than typically measured in similar systems (above $2\mu\text{F}\cdot\text{cm}^{-2}$ for monolayer $MoSe_2$[10] and WSe_2[11]), which might be related to a work function mismatch between ReS_2 and the PE.[4] The carrier density at V_{PE} = 0V is on the order of 10^{12} cm^{-2}, which might originate from residual doping due to intrinsic defects. Our trilayer ReS_2 device has a back gate coated with 270 nm SiO_2. On Supplementary Figure 8b, we plot carrier densities at each cooldown as a function of V_{bg}. The extracted back gate capacitance for each cooldown corresponds very well to the geometric capacitance, showing that our measurements are correct. On Supplementary Figure 8c the full set of mobility data is presented. The overall decrease of Hall mobility is evident with the increase of V_{PE}.

For all measurements, an effort was made to obtain a linear reading from the voltage probes: in the more resistive state, the temperature and gate voltage were increased until a reliable measurement was obtained (Supplementary Figure 8b).

Supplementary Figure 8(a) also contains information on the ionic concentration n_i in units of amount of ions per unit cell of ReS_2 (we extract it with the assumption that 1 electron in the conduction band corresponds to one positive ion on top of the conducting channel, conversion coefficient 2.875×10^{14}). As shown on Figure 5 of the main text, our experimentally obtained

carrier densities and ionic concentrations are very close to those obtained from theoretical modeling.

We notice that for the case of monolayers, the systematic Hall-effect data and dependence of carrier density on V_{PE} was difficult to extract. We observed a purely insulating behavior (decreasing conductivity with decreasing temperature) for all carrier densities in monolayer ReS_2 EDLT. According to studies[12,13] of systems with similar transport regimes, Hall-effect data might not give a reliable carrier density in this case. Additional complications might originate from noise, which is a common feature of transport in hopping regimes[13,14]. Looking at similar experiments[10,11] on other monolayer and multilayer 2D materials gated by [EMIM]-[TFSI], we would expect that the gate capacitance and maximum achieved carrier density should be roughly the same in mono-and multi-layer ReS_2 near the conductivity dome.

Supplemenraty Note 8: Band structure calculation for bulk ReS_2 and effective mass calculations for monolayer and bulk ReS_2

The calculated electronic band structure of bulk ReS_2 is shown in Supplementary Figure 9. It differs from the one calculated for monolayer ReS_2 in a smaller energy gap E_g = 0.92 eV (1.35 eV for monolayer ReS_2). The bulk gap appears at the Z point, which coincides with the Γ point upon a projection of the three-dimensional BZ onto the $k_x k_y$ plane.

The effective mass tensors are obtained by calculating the energy bands on a fine *k*-point grid (mesh spacing of 0.005 in units of 2π/a, where *a* is the lattice constant along the zigzag direction) centered around the Γ point (the Z point for bulk), and by calculating the derivatives using finite differences to obtain the inverse effective mass tensor.

In the following, the *x*-direction is defined as the direction along the zigzag chains, the *y*-direction is in-plane, perpendicular to *x*, and the *z*-direction is perpendicular to the *xy* plane. The mass tensor for the bulk crystal, $M^Z_{i,j=\{x,y,z\}}$, f in units of the electron mass, is

M^Z

0.42	0.04	0.05
0.04	0.47	0.23
0.05	0.23	1.09

whereas the mass tensor for 1L ReS_2, $M^Z_{i,j=\{x,y\}}$, is

M^X

0.50	0.07
0.07	0.91

From diagonalization of the effective mass tensor for the monolayer, we find that m^* reaches the minimum value $m^*_L = 0.48\ m_e$, where m_e is the electron mass, along a "light" direction that forms an angle $\gamma_m = 9.5°$ with respect to a Re atom chain, whereas along the orthogonal "heavy" direction we find $m^*_H = 0.92\ m_e$. The calculated anisotropy ratio $m^*_L/m^*_H = 0.53$ is very close to the one measured for conductivity.

Supplementary Note 9: Variable range hopping in multilayer ReS_2

We extracted the localization length from our measurements, using the variable-range hopping (VRH) model. We used the expression $\xi_{\mathrm{loc}} = \sqrt{13.8 / k_{\mathrm{B}} D T_0}$, where $D(E)$ - is the density of states in the conduction band of ReS_2 and $T_0 = 3\times10^5\,\mathrm{K}$ - could be extracted from the slope of $\ln R$ as a function of $T^{-1/3}$. The same analysis performed on another device resulted in $T_0 = 9.7\times10^4\,\mathrm{K}$. We calculated an effective mass of ReS_2 $m^* = 0.5\ m_0$ (Supplementary Note 10) and extracted the density of states using $D = g_s \times g_v \times m^* / \pi\hbar^2$. We found $D_{\mathrm{2D}}^{\mathrm{theory}} = 4.17\times10^{14}\,\mathrm{eV^{-1}cm^{-2}}$ and $\xi_{\mathrm{loc}} \approx 0.35 \div 0.63\,\mathrm{nm}$.

Taking into account the carrier densities extracted from the Hall effect data, we could estimate the imbalance between positive and negative ions. At $V_{\mathrm{PE}} = 3.4$ V the average distance between the positive ions would be below 1.6 nm. Although the ions could form different types of clusters[15] on the surface, even this simple approximation results in the same order of magnitude as our localization length.

Supplementary Note 10: Multiphonon hopping in multilayer ReS_2

In this section, we discuss the possibility of multiphonon hopping in multilayer ReS_2 for the device discussed on Figure 4. The multiphonon hopping regime (MPH) has a power law dependence of conductivity on temperature $R \propto R_0(T/T_0)^p$. The exponent $p = -5$ could be extracted from the average value of the plateau (Figure 4(a)). It corresponds to the number of phonons that electron exchanges energy with an electrons as it hops to its next location.[16] The temperature range for this distinctive plateau is 50 − 96 K. MPH is a typical feature of amorphous and disordered systems at low temperatures,[16–20] but we do not exclude the possibility of such behaviour in multilayer ReS_2 at high n_{2D}. We are also aware of the fact that a clear distinction between two mechanisms might not be always possible due to similarities in the temperature dependence.[16] We notice, that in the work of Xie et al.[21] MPH in rubrene EDLT was observed after a similar mobility peak in the valence band of rubrene but without a complete conductivity suppression like in monolayer ReS_2.

Supplementary Note 11: Alternative explanations of conductivity suppression at high carrier densities in ReS_2

Extensive investigation allowed us to exclude alternative explanations for the observed conductivity suppression at high doping densities: structural phase transition due to doping, Fermi level reaching the dip in the density of states (DOS) upon doping due to the complete filling of the narrow conduction band, band structure modifications induced by the transverse electric field generated by the electrolyte gate.

Doping influence on anisotropy in monolayer ReS_2. The lattice distortion in ReS_2 leads to anisotropic transport: the material is more conductive along the direction of the Re atomic chains (see scanning transmission electron micrograph on Supplementary Figure 7a).[7] High doping levels could result in lattice distortion[22] and modify electrical conductivity in different directions. To examine this, we looked at the conductivity anisotropy, using directional devices with eight electrical contacts rotated by 45° (Supplementary Figure 10). These experiments revealed that the ratio between conductivities in the range of carrier densities that allows us to reliably perform such measurements stays within the same sinusoidal dependence and corresponds well to both early results on bulk ReS_2[23] and recent monolayer measurements.[7] Additionally, we found good agreement between experimentally measured anisotropy and

theoretically calculated effective masses. We notice though, that at high doping levels the curves merge and become undistinguishable. This is in a good agreement with our transport model.

Possibility of a phase transition due to doping. We have performed structural optimization calculations, for different levels of doping, on various supercells of ReS_2. Our calculations did not reveal any phase other than the 1T′ phase with 2×2 periodicity that always appears to be the energetically most favorable. Moreover, we verified that doping does not significantly alter the energy bands, except for the position of the Fermi level.

Filling of the narrow conduction band. This explanation is in principle supported by the almost perfect disentanglement of the first conductance band and correspondingly, the presence of a partial suppression in the DOS, see Figure 5(c) and Supplementary Figure 9c. Nevertheless, complete filling of the band translates into adding exactly 2 extra electrons per unit cell (including spin degeneracy), whereas the highest charge concentration measured in experiments is about 0.08 electrons per unit cell (2.3×10^{13} cm^{-2}), thus ruling out this hypothesis.

Electric field influence on the band structure. We have performed band structure calculations within DFT for monolayer ReS_2 including a perpendicular electric field with intensity varying in the 1 − 8 V×nm^{-1} range. We found only minor changes in the low-energy region with respect to the energy bands of the unperturbed system, shown in Figure 5. Therefore, we exclude the electric field as a source of the observed conductivity decrease.

Supplementary Note 12: Transport model for monolayer ReS_2

The unit cell of the lattice of monolayer ReS_2 has been mapped onto a rectangular lattice, with x and y direction corresponding, respectively to the easy and hard axes for the electronic transport. The lattice vectors a_x = 6.37 Å and a_y = 5.68 Å ensure conservation of the unit cell surface area. We describe the lowest conduction band of ReS_2, highlighted in Figure 5(c), via a tight-binding model that considers one orbital per atom and next-neighbor interaction:

$$\hat{H} = \sum_{i\neq j} t_{ij} a_i^\dagger a_j + \sum_i \varepsilon_i a_i^\dagger a_i \qquad (4)$$

with

$$t_{i,j} = \begin{cases} t_x & \mathit{if} \quad \mathbf{r}_i - \mathbf{r}_j = (\pm a_x, 0, 0) \\ t_y & \mathit{if} \quad \mathbf{r}_i - \mathbf{r}_j = (0, \pm a_y, 0) \\ 0 & \mathit{otherwise} \end{cases} \qquad (5)$$

The hopping parameters $t_{x(y)}$ have been chosen in order to match the effective masses of the model with that calculated from *ab initio*, $t_{x(y)} = \hbar^2 / (2m^*_{x(y)} a^*_{x(y)})$, yielding t_x = -0.187 eV and t_y = −0.130 eV.

The electric double layer at the interface between the ionic liquid and the semiconductor has been modeled by placing monovalent ions (e.g. Li^+) at a distance Δz = 20 Å from the plane of the rectangular lattice. When no gate voltage is applied, an equal number of positive and negative ions are in proximity of the semiconductor, with the total ionic charge vanishing. Therefore, ions are arranged initially in a bipartite square lattice with periodicity D_{ion} = 10 Å, such that two neighboring ions have opposite charge. An applied voltage is responsible for an imbalance between the number of positive and negative ions, N_+ and N_-, respectively. We model this effect by flipping the sign of a certain amount of randomly selected ions. In order

to guarantee the total charge neutrality of the ionic gate-semiconductor interface, the concentration of imbalanced ions, defined as $n_{ions} = (N_{+}-N_{-})/N_{cells}$ must equal the concentration of induced electrons $n_{el} = N_{el}//N_{cells}$. The quantitative equivalence of n_{ions} and n_{el} is a key feature of our model.

The presence of an electrostatic potential is encoded into the model by adding non-zero on-site energies calculated from the random distribution of ions. For the i-th site, at position $\mathbf{r}_i$, the potential on-site energy is

$$\varepsilon_i = \frac{e^2}{4\pi\varepsilon_0\varepsilon_r}\left(\sum_j^{N_-}\frac{1}{|\mathbf{r}_i-\mathbf{R}_j|}-\sum_j^{N_+}\frac{1}{|\mathbf{r}_i-\mathbf{R}_j|}\right)e^{-(|\mathbf{r}_i-\mathbf{R}_j|/r_0)} \tag{6}$$

where $\mathbf{R}_j$ is the position of the j-th ion, $\varepsilon_r = 37$ is the relative dielectric constant of acetonitrile, and $r_0 = 20$ nm is a screening length of the order of magnitude of the Debye length in doped semiconductors. As shown in Supplementary Figure 11, the mean value of the electrostatic energy $<\varepsilon_i>$ decreases linearly as a function of n_{ions} as expected for an effective planar capacitor. However, the amplitude of the spatial fluctuations of the potential energy increases with doping, as shown in Supplementary Figure 11, resulting in a stronger electrostatic disorder.

The electric conductivity has been calculated by means of the Kubo formula[24,25] assuming a linear response to the applied electric field.

The regular part of DC longitudinal conductivity, is thus given by

$$\sigma_{i,i} = \lim_{\omega\to 0^+}\frac{\pi(1-e^{-\beta\hbar\omega})}{S\hbar\omega}\sum_{m\neq n}p_m(1-p_n)\left|\left\langle m\left|\hat{J}_a\right|n\right\rangle\right|^2\delta(E_m-E_n-\hbar\omega) \tag{7}$$

where β is the inverse temperature $1/k_BT$, S is the area of the model supercell, $\hat{J}_a$ is the current operator along direction $a = x,y,$ $|m\rangle$ is a generic eigenstate with energy E_m occupied with a probability $p_m = 1/(1+e^{\beta(E_m-\mu)})$, with μ being the chemical potential determined by the electron concentration n_{el}.

The current operator for a rectangular tight-binding model is defined as

$$\hat{J}_a = -\frac{ie}{\hbar}\sum_{i\neq j}t_{i,j}(\mathbf{R}_i-\mathbf{R}_j)_a a_i^\dagger a_j \tag{8}$$

The Dirac delta appearing in Equation (7) is replaced by a Lorentzian function

$$\delta(E_m-E_n+\hbar\omega)\leftarrow\frac{1}{\pi}\frac{\eta}{(E_m-E_n+\hbar\omega)^2+\eta^2} \tag{9}$$

where the broadening $\eta = 1$ meV is related to the phase coherence time $\tau_\phi \simeq \hbar/\eta \simeq 7\times10^{-13}$s.

Supplementary Methods

Crystal growth. Single crystals of ReS_2 were grown using the chemical-vapor transport (CVT) method, using Br_2 or ICl_3 as the transport agent. Prior to crystal growth, quartz tubes containing the elements (Re, 99.95% pure; S, 99.999%) with Br_2 or ICl_3 were evacuated and sealed. The

quartz tube was placed in two zones of a three-zone furnace and the charge was pretreated for 24 h at 800°C with the growth zone at 1000°C, preventing the transport of the product. The furnace was then equilibrated to give a constant temperature across the reaction tube, and was programmed over 24 h to give the temperature gradient at which single-crystal growth took place. Best results were obtained with temperature gradients of approximately ΔT = 150°C = 1160°C −1010°C. ReS_2 formed silver-colored, graphite-like, thin hexagonal platelets up to 2 cm^2 in area and 100 µm in thickness. Additionally, we used commercially available crystals from HQ Graphene (http://www.hqgraphene.com/). We did not observe significant differences in terms of doping and observed the insulating state at high carrier densities in samples from both sources.

Device fabrication. Devices of single- and multilayer ReS_2 were obtained from bulk crystals, which were cleaved onto degenerately n++ doped Si covered with 270 nm SiO_2. Thickness, measured by AFM, was correlated with optical contrast. The contacts were obtained with standard electron beam lithography followed by electron beam evaporation of Pd-Au (5-50 nm) stack and liftoff in acetone. A second step of electron-beam lithography was used to define the etching mask. Etching was performed with O_2 or SF_6/O_2 plasma depending on the flake thickness. A series of devices were fabricated by transferring monolayer flakes on top of local back gates (Cr/Au 10/30 nm) covered with 30 nm HfO_2, grown by atomic layer deposition (ALD), which gives access to higher carrier densities in comparison with 270 nm SiO_2. Contacts and electrodes for polymer electrolyte voltage were exposed at the same lithography step. Electrode for polymer electrolyte (PE) gating had >5 times bigger area than contacts. We have performed the measurements on two types of devices in the case of monolayers: directional devices with 8 contacts laid out every 45 degrees and Hall-bar structures for sheet conductivity measurements.

Ion gel preparation. In this work, 3 main recipes for ion gels were used. Most of the devices were measured based on the PS-PMMA-PS:[EMIM]-[TFSI] ion gel:[26,27] EMIM-TFSI (918 mg), PS-PMMA-PS (68 mg) prepared in anisole (8874 mg). The mixture was stirred for 5h at room temperature. Several devices were measured with PE based on $LiClO_4$: $LiClO_4$ (302.4 mg) and polyethylene oxide (PEO) (1009 mg) dissolved in 15 ml of methanol[28] or 1:8 mixture of $LiClO_4$ and PEO in acetonitrile (10% weight). We did not see any differences in performance, see Supplementary Figure 2 for details and comparison.

Electrical and cryogenic measurements. All electrical and cryogenic measurements were performed in vacuum after annealing. For devices with the polymer electrolyte, we used 70 ºC annealing for 2 hours. For the devices with solid back gate, we annealed for several hours at 140 ºC to remove absorbed water, O_2 and possible contamination, which could mask the intrinsic properties of ReS_2. We used four-probe geometry to extract the sheet resistivity G of ReS_2 flakes (see Figure 2(d)). The sweep rate of polymer electrolyte voltage was varied from 0.2 to 50 mV/s, depending on the device. We did not observe any strong dependence of device performance on the sweep rate (Figure 1 and Supplementary Figure S3) except for a higher leakage (charging) current at higher speeds, which in all our experiments was kept below 2 nA. For the devices discussed in the main text, the V_{PE} is applied at room temperature, the current is stabilized and the cooldown is performed with constant speed (1K/min). Freezing point of PS-PMMA-PS:[EMIM]-[TFSI] was established to be between 180K and 230K, where the device stopped reacting on the application of V_{PE}. Below that point, we could use the back gate to modulate the carrier density around the value set using the PE. For DC electrical measurements we used Agilent 5270B SMU and Keithley 2000 DMM. Cryogenic measurements were performed in a Janis closed-cycle cryogen-free cryostat. For Hall effect

measurements we used an Oxford instruments Heliox system with a superconducting magnet (±10T).

Scanning Transmission Electron Microscopy (STEM) imaging. Aberration-corrected scanning transmission electron microscopy (STEM) was performed using an FEI Titan Themis 60−300 transmission electron microscope operated with a beam energy of 80 keV. Medium angle annular dark field (MAADF) imaging conditions were applied with a probe convergence semi-angle of ~28 mrad and a STEM detector collection semi-angle of ~40−200 mrad. The probe current was kept below 50 pA. We used the sequence of images, acquired with low doses to avoid flake damage, and furthermore aligned and averaged using Digital Micrograph or ImageJ.

Computational methods for first principles calculations. Electronic structure calculations presented in this work were performed using the Quantum-ESPRESSO package[29] with projector-augmented wave potentials[30]. We used the generalized gradient approximation (GGA) according to Perdew, Burke and Ernzerhof (PBE) exchange-correlation functional.[31] A plane-wave kinetic energy cutoff of 53 Ry (438 Ry for charge density) and a Γ-centered 6×6×1 (6×6×6) mesh for k-point sampling in the case of monolayer (bulk) calculations were used. We include both 5s and 5p semi-core states in the valence for Re atoms. Except for the cell geometry optimization where only scalar relativistic corrections were used, all calculations were carried out taking into account spin-orbit coupling. The convergence thresholds for ionic relaxations are such that all components of all forces are smaller than 10^{-4} a.u. The criterion for the unit cell geometry optimization is that the pressure is less than 0.05 kbar.

Supplementary References

1. Dumcenco, D. *et al.* Large-Area Epitaxial Monolayer MoS_2. *ACS Nano* **9,** 4611–4620 (2015).
2. Allain, A. & Kis, A. Electron and Hole Mobilities in Single-Layer WSe_2. *ACS Nano* **8,** 7180–7185 (2014).
3. Ye, J. T. *et al.* Superconducting Dome in a Gate-Tuned Band Insulator. *Science* **338,** 1193–1196 (2012).
4. Shi, W. *et al.* Superconductivity Series in Transition Metal Dichalcogenides by Ionic Gating. *Sci Rep* **5,** (2015).
5. Yuan, H. *et al.* Zeeman-type spin splitting controlled by an electric field. *Nat Phys* **9,** 563–569 (2013).
6. Costanzo, D., Jo, S., Berger, H. & Morpurgo, A. F. Gate-induced superconductivity in atomically thin MoS_2 crystals. *Nat. Nanotechnol.* **11,** 339–344 (2016).
7. Lin, Y.-C. *et al.* Single-Layer ReS_2: Two-Dimensional Semiconductor with Tunable In-Plane Anisotropy. *ACS Nano* (2015).
8. Shibata, H. & Oide, J. Analysis of the Hall effect device using an anisotropic material. *J. Appl. Phys.* **88,** 4813–4817 (2000).
9. W L V Price. Extension of van der Pauw's theorem for measuring specific resistivity in discs of arbitrary shape to anisotropic media. *J. Phys. Appl. Phys.* **5,** 1127 (1972).
10. Chang, Y.-H. *et al.* Monolayer $MoSe_2$ Grown by Chemical Vapor Deposition for Fast Photodetection. *ACS Nano* **8,** 8582–8590 (2014).
11. Huang, J.-K. *et al.* Large-Area Aiming Synthesis of WSe_2 Monolayers. *ACS Nano* **8,** 923–930 (2014).
12. Friedman, L. & Pollak, M. Hall mobility due to hopping-type conduction in disordered systems. *Philos. Mag. Part B* **38,** 173–189 (1978).
13. Podzorov, V., Menard, E., Rogers, J. A. & Gershenson, M. E. Hall Effect in the Accumulation Layers on the Surface of Organic Semiconductors. *Phys. Rev. Lett.* **95,** 226601 (2005).
14. Lee, B. *et al.* Trap healing and ultralow-noise Hall effect at the surface of organic semiconductors. *Nat. Mater.* **12,** 1125–1129 (2013).
15. Xie, W., Liu, F., Shi, S., Ruden, P. P. & Frisbie, C. D. Charge Density Dependent Two-Channel Conduction in Organic Electric Double Layer Transistors (EDLTs). *Adv. Mater.* **26,** 2527–2532 (2014).
16. Shimakawa, K. Multiphonon hopping of electrons on defect clusters in amorphous germanium. *Phys. Rev. B* **39,** 12933–12936 (1989).

17. Zvyagin, I. P. Multiphonon hopping in disordered semiconductors. *Sov. Phys. J.* **19,** 162–167 (1976).
18. Somaditya Sen and A Ghosh. Multiphonon assisted hopping in strontium vanadate semiconducting glasses. *J. Phys. Condens. Matter* **11,** 1529 (1999).
19. Bhattacharya, S., Chaudhuri, B. K. & Sakata, H. Multiphonon hopping conduction in nonconventional chromium-doped Bi3Pb1Sr3Ca3Cu4−nCrnOx (n=0.025–0.2) glasses with nanocrystalline particles and clusters. *J. Appl. Phys.* **88,** 5033–5042 (2000).
20. Serin, T., Yildiz, A., Şahin, Ş. H. & Serin, N. Multiphonon hopping of carriers in CuO thin films. *Phys. B Condens. Matter* **406,** 3551–3555 (2011).
21. Xie, W., Wang, S., Zhang, X., Leighton, C. & Frisbie, C. D. High Conductance 2D Transport around the Hall Mobility Peak in Electrolyte-Gated Rubrene Crystals. *Phys. Rev. Lett.* **113,** 246602 (2014).
22. Okuyama, D. *et al.* Gate-tunable gigantic lattice deformation in VO2. *Appl. Phys. Lett.* **104,** (2014).
23. Ho, C. H., Huang, Y. S. & Tiong, K. K. In-plane anisotropy of the optical and electrical properties of ReS_2 and $ReSe_2$ layered crystals. *J. Alloys Compd.* **317–318,** 222–226 (2001).
24. Kubo, R. Statistical-Mechanical Theory of Irreversible Processes. I. General Theory and Simple Applications to Magnetic and Conduction Problems. *J. Phys. Soc. Jpn.* **12,** 570–586 (1957).
25. Gargiulo, F., Perroni, C. A., Ramaglia, V. M. & Cataudella, V. Electronic transport within a quasi-two-dimensional model for rubrene single-crystal field effect transistors. *Phys. Rev. B* **84,** 245204 (2011).
26. Pu, J. *et al.* Highly Flexible MoS_2 Thin-Film Transistors with Ion Gel Dielectrics. *Nano Lett.* **12,** 4013–4017 (2012).
27. Yomogida, Y. *et al.* Ambipolar Organic Single-Crystal Transistors Based on Ion Gels. *Adv. Mater.* **24,** 4392–4397 (2012).
28. Yu, Y. *et al.* Gate-tunable phase transitions in thin flakes of 1T-TaS2. *Nat Nano* **10,** 270–276 (2015).
29. Giannozzi, P. *et al.* QUANTUM ESPRESSO: a modular and open-source software project for quantum simulations of materials. *J. Phys. Condens. Matter* **21,** 395502 (2009).
30. Kresse, G. & Joubert, D. From ultrasoft pseudopotentials to the projector augmented-wave method. *Phys. Rev. B* **59,** 1758–1775 (1999).
31. Perdew, J. P., Burke, K. & Ernzerhof, M. Generalized Gradient Approximation Made Simple. *Phys. Rev. Lett.* **77,** 3865–3868 (1996).
